# Application of 5G Internet of Things Technology in the Design of Physical Education Platform

**DOI:** 10.1155/2022/9382048

**Published:** 2022-04-21

**Authors:** Jing Wang

**Affiliations:** School of Physical Education, Zheng Zhou Sias University, Xinzheng, Henan 451150, China

## Abstract

The traditional college physical education platform can only assist classroom teaching and cannot collect classroom teaching activity data in real time, and its role is very limited. With the rapid development of information technology, China's mobile communication has entered the 5G era and the Internet of Things technology will be more widely used and developed. This paper focuses on the integration technology of 5G and the Internet of Things, analyzes the current situation of the construction of the physical education platform in colleges and universities, and proposes the application of the 5G-based Internet of Things technology in the physical education platform. At the same time, the research of this paper is oriented to real-time physical education activities, using the Internet of Things technology and wireless network technology, combined with the traditional campus network and IT information technology means, to propose a new, dynamic, and open sports network teaching platform design. Activity data collection, analysis, modeling, mining, display, feedback, self-improvement, etc. are expounded. At the same time, the realization technology of the model is discussed, and the experimental design of the combination of the platform and volleyball sports teaching is carried out. The experiment shows that through the platform, simulation can better promote students' understanding, mastery, and application of basic techniques such as passing, padding, and serving. The research results show that the platform can make the teaching content intuitive and collect teaching activity data in real time. The interaction between students is helpful for teachers to strengthen the guidance of students' learning process and improve the quality of physical education in higher vocational education.

## 1. Introduction

Since its birth in the 1980s, mobile communication technology has experienced explosive development for more than 30 years. Today, wireless communication networks have been integrated into every aspect of people's lives and have had a profound impact on the social environment and economic model. With the popularization of 4G large-scale wireless communication technology and the development of emerging technologies, people have put forward more demands on mobile communication networks. The emergence of the Internet of Things (IoT) technology has allowed more hardware devices to connect to the base station network, which has increased the data traffic of the base station network by multiple orders of magnitude. Some experts predict that, by 2020, there will be hundreds of billions of mobile communication devices and more diversified mobile network application services in the world, and wireless communication traffic will show a thousandfold increase in the next decade. It poses great challenges in terms of resources and transmission delay. Therefore, there is an urgent need to promote the development of a new generation of mobile communication networks. 5G mobile communication technology has become a hot research topic for researchers in recent years [1–7].

**Figure 1:**
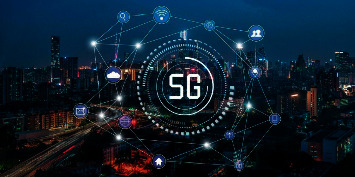
5G IoT.

As the main driving force for the development of mobile communication networks, IoT technology has made mobile data business scenarios increasingly diversified. Data services are no longer limited to text, voice, and video, but are expanding to remote control and intelligent systems. Facing the rich application scenarios of 5G mobile networks, the International Telecommunication Union (ITU) has classified them: enhanced mobile broadband (eMBB) scenarios and massive machine type communications (mMTC) scenarios and low-latency and high-reliability (ultrareliable and low-latency communications, uRLLC) scenarios. Enhanced mobile broadband scenarios are mainly for bandwidth-sensitive services, such as high-definition video, virtual/augmented reality (VR/AR); massive device communication scenarios are mainly for connection-intensive services, such as intelligent transportation and smart factories; low-latency and high-reliability communication scenarios are mainly for latency-sensitive services, such as autonomous driving and remote control. To give a specific example, shared bicycles are a product brought about by the development of mobile communication technology—Mobikes are connected end to end under the cellular mobile network, and OFO bicycles are equipped with NB-IoT to access the base station network. It can be seen that the shared bicycle is one of the three scenarios defined by 5G—the actual case of the communication scenario of massive devices—and this is just the beginning. The low-latency and high-reliability scenario is the key to 5G commercial use. Therefore, optimizing the mobile communication network, reducing the delay, and improving the transmission reliability are among the key issues that need to be solved urgently in the evolution of 5G [8–14], as shown in Figure 1.

With the rapid development of technology and economy, IoT technology has begun to be widely used in various industries. With the opening of the 5G era, the connection between mobile communication technology and the Internet of Things has been further expanded. The integration of the two can greatly promote the development of science and technology and greatly facilitate people's lives. At the same time, whether it is from the requirements of the national development strategy or from the actual needs of the school itself, the construction of smart campuses is of great significance and has become an inevitable trend in the development of colleges and universities [15–20].

5G adopts key technologies, such as massive MIMO, ultradense networking (UDN), full-spectrum access, new multiple access, new multicarrier, and terminal direct connection to build a powerful basic bearer network with the characteristics of low latency, high reliability, and large throughput, provide basic network support for the Internet of Everything. The fusion of the two specifically has the following advantages:

1. High speed: 5G has a large capacity, and every smart device can be directly connected to a 5G base station. It makes the transfer rate greatly accelerated.
2. Convenience: 5G-based network deployment and optimization can utilize existing cabling without requiring large-scale demolition and construction, making the implementation process more convenient. On the other hand, 5G uses millimeter-wave communication to reduce the size of communication equipment, and the exchange of information between equipment is more convenient.
3. Economy: IoT devices are directly connected to 5G mobile phones through the network, reducing the use and cost of devices such as ZigBee, routers, and switches.
4. Safety: 5G has the authentication and encryption technology, which can effectively improve the stability and security of data transmission on the basis of improving the communication speed.

In order to improve the teaching mode of the physical education auxiliary platform in colleges and universities, using the Internet of Things technology and wireless platform technology, based on the original campus network, this paper proposes an idea of building a new teaching auxiliary information platform for college sports based on the Internet of Things and mobile platform technology. It provides a new attempt to realize the use of advanced information technology to assist physical education teaching. Traditional college physical education platforms are usually implemented based on Web development technology, based on teaching resources and background databases, and are generally displayed in the form of teaching video resources and HTML. Although the traditional higher vocational physical education platform can present the teaching content intuitively and vividly, it is beneficial to better display the relevant theoretical knowledge, teaching content, and exercise process for students and has a positive effect on stimulating students' interest in learning and improving the effect of higher vocational physical education teaching. However, this kind of teaching platform is only a supplement to classroom teaching. It cannot collect real-time and detailed data of higher vocational physical education classroom teaching activities, and it is also difficult to use advanced technologies such as Internet of Things technology and mobile terminal technology to conduct data mining analysis and simulation modeling and use it to test and improve vocational physical education [21–25].

Based on IoT sensor technology, remote sensing technology can transform students or teachers in teaching activities into “active points,” which is equivalent to digitally storing the movement process for data preparation for future simulation, analysis, and modeling, which lays a data foundation for reproducing the actual teaching activity model. The change of this model is a new change compared to the original teaching model.

Originating from actual teaching activities, evaluating and improving actual teaching activities, with theoretical models and actual dynamic models, a scientific and reasonable evaluation index system can be formulated according to the actual situation, which can be used to guide and evaluate the teaching of each classroom. Activities can earnestly grasp the important indicators and data in teaching activities, speak with actual data, and formulate improvement plans based on actual data, which is of great significance to effectively guiding each student and each teaching activity great help.

In general, with the increasing development of network and information technology, especially the development of Internet of Things and mobile platform technology, new opportunities and challenges have been brought to the original information platform. It also provides new technical opportunities for universities to run these technologies to assist teaching and research. In order to make up for the above deficiencies, let the teaching platform better assist the development of higher vocational physical education teaching activities, and also in order to improve the mode of the existing university physical education platform, this paper applies the Internet of Things technology and wireless platform, relying on the original campus network, and proposes a method based on the Internet of Things. The design idea of building a new university physical education platform system based on networking and mobile platform technology provides a new model for the realization of information technology-assisted physical education teaching. Through the application of Internet of Things sensor technology, remote sensing technology, etc., the platform can collect the information of physical education activities in colleges and universities in a timely manner and store it in a digital way. At the same time, the construction of the platform's dynamic self-feedback system is conducive to evaluating, improving, and perfecting the university system.

## 2. Communication Algorithm

This algorithm adopts a simplified energy model widely used in scientific research. The power loss channel model will be used for short distance transmission and the power loss channel model will be used for long distance transmission. Formula (1) represents the energy consumed by the transmitting node to send a data packet with a length of 1 bit to a receiving node beyond *d* distance, and formula (2) represents the energy consumed by the receiving node to receive a data packet with a length of 1 bit.

(1) $$\begin{array}{c} E_{Tx}\left(l,d\right)=\left\{\begin{array}{c} lE_{\text{elec}}+l\varepsilon_{fs}d^{2}\;d<d_{0}\\ lE_{\text{elec}}+l\varepsilon_{mp}d^{4}\;d\geq d_{0} \end{array}\right). \end{array}$$

(2) $$\begin{array}{c} E_{Rx}\left(l,d\right)=lE_{\text{elec}}, \end{array}$$

where *E* is the energy consumption caused by signal processing in the transceiver circuit, which is related to factors such as coding, modulation, and filtering. *ε* is the power consumption amplification factor, which is related to the transmission distance and the acceptable bit error rate. The distance threshold can be expressed as

(3) $$\begin{array}{c} d_{0}=\sqrt{\frac{\varepsilon_{fs}}{\varepsilon_{mp}}.} \end{array}$$

For family member nodes, each cluster member node only sends data packets to its corresponding cluster head node. In each time slot, the energy consumed by the cluster member nodes in the kth layer is

(4) $$\begin{array}{c} E_{k}=lE_{\text{elec}}+l\varepsilon_{fs}E\left[d^{2}\right], \end{array}$$

where *d* is the square of the distance from the cluster member node in the kth layer to its corresponding cluster head node. The expected value of *E* can be expressed as

(5) $$\begin{array}{c} E\left[d^{2}\right]=\int_{0}^{2\pi }\int_{0}^{\sqrt{2k-1/m_{k}}}\rho r^{3}dr\;d\theta ,\\ \int_{0}^{2\pi }\int_{0}^{\sqrt{2k-1/m_{k}}}\rho r^{3}dr\;d\theta =\frac{2k-1}{2m_{k}}r^{2}. \end{array}$$

For cluster head nodes, the following formula gives the energy consumption of each cluster head node:

(6) $$\begin{array}{c} E_{k}=lE_{elec}\left(\frac{n_{k}}{m_{k}}-1\right)+lE_{DA}\frac{n_{k}}{m_{k}}+l\varepsilon_{fs}E\left[d^{2}\right]+lE_{elec}+l\frac{\sum_{i=k+1}^{k}m_{i}}{m_{k}}\left(E_{elec}+\varepsilon_{fs}E\left[d^{2}\right]\right), \end{array}$$

where n_k_ is the total number of nodes in the k-th layer, which is represented as

(7) $$\begin{array}{c} n_{k}=\frac{2k-1}{K^{2}}N. \end{array}$$

The expected values are given in the following equations, respectively:

(8) $$\begin{array}{c} E\left[d^{2}\right]=\frac{k^{2}+{\left(k-1\right)}^{2}}{2}r^{2}. \end{array}$$

(9) $$\begin{array}{c} E\left[d\right]=\frac{2\left[k^{3}+{\left(k-1\right)}^{3}\right]}{3\left(2k-1\right)}r. \end{array}$$

A cluster in the kth layer consists of a cluster head node and a cluster member node. Therefore, the total energy consumption in a cluster is the sum of the energy consumption of the cluster head node and the energy consumption of all cluster member nodes, which can be expressed as

(10) $$\begin{array}{c} E_{k}^{\text{cluster}}=E_{k}^{CH}+\frac{n_{k}}{m_{k}}E_{k}. \end{array}$$

The total energy consumption in the k-th layer is the sum of the energy consumption of all clusters in this layer:

(11) $$\begin{array}{c} E_{k}=lE_{elec}+lE_{DA}n_{k}+m_{k}l\varepsilon_{fs}E\left[d^{2}\right]+\sum_{i=k+1}^{K}m_{i}l\left(E_{elec}+\varepsilon_{fs}E\left[d^{2}\right]\right). \end{array}$$

## 3. Design of the Sports Information Teaching Platform

Aiming at the intelligent management of physical education courses, a service-oriented Internet of Things architecture is used to build a physical education course management system architecture based on the Internet of Things. The architecture includes client, client, application support, application, infrastructure, and server.

The infrastructure and server manage the information resources of sports courses and feed back the information to the application side through the Internet of Things. The application side completes the arrangement, selection, and information exchange of sports courses according to the information. The wireless network feeds back the sports course management results on the application side to the teachers and students on the user side through the client's PC browser, mobile phone browser, and other interactive interfaces, providing services for teachers and students to arrange and select courses reasonably.

The system users are students and teachers. Students and teachers usually use PC browsers, mobile browsers, tablet browsers, or touch screens as clients to browse and set up physical education courses in the system. The internal application support side of the system includes transaction processing, Internet, wireless network, protocol between sensor networks, XML exchange, and data engine. It aggregates these information resources and provides an integration that supports information access, transmission and collaboration through a unified access portal. In this environment, teachers and students can browse the interrelated data of physical education course management and process the relevant content of physical education course management according to the related data. The application side includes four parts: teacher course arrangement module, student course selection module, student basic information setting module, and interactive communication module. Infrastructure and services should be viewed from the two aspects of facilities and services. Basic services include data collection, data storage, data synchronization, basic management, and data backup in sports course management; infrastructure includes servers, storage, networks, query platforms, and information. Management: the information management part manages information through a physical education course information management model based on the Internet of Things and transmits the information to the server. In order to better understand the functions of the application side in the architecture of the sports course management system based on the Internet of Things, the following is a detailed analysis of the four modules included in the application side.

**Figure 2:**
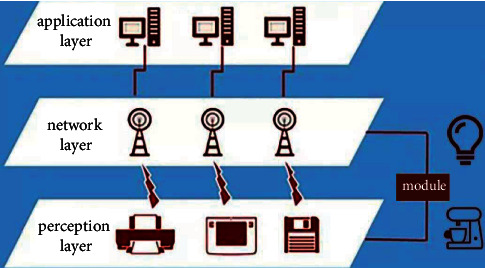
Three-layer IoT.

The Internet of Things is an interconnected network of things and things, which consists of a perception layer, a network layer, and an application layer (see Figure 2). Its foundation and core are still the Internet, a network that extends and expands on the basis of the Internet. At the same time, through the extension and expansion of the network, information exchange and communication can be realized between objects. It is precisely because the Internet of Things has significant characteristics and advantages in this respect that it can effectively meet the needs of teaching platform design, so it is more and more widely used in teaching platform design.

The distinctive features of physical education activities in colleges and universities are practical and dynamic. Through the application of Internet of Things technology, combined with the basic situation of physical education in colleges and universities, a new teaching platform is constructed, which is conducive to better auxiliary teaching, facilitates data collection, storage, and monitoring of the entire teaching process of physical education in colleges and universities, and can grasp the dynamics of physical education teaching in detail and comprehensively. Data, and take improvement measures for existing deficiencies, will help improve the quality of physical education in colleges and universities.

The design of college physical education platform based on the Internet of Things cannot be separated from the support of advanced technology. Therefore, in the design, according to the needs of college physical education, closely focus on the inherent characteristics of the platform, give full play to the role of advanced technology, and build a layered platform model. The platform construction mainly applies the following advanced technologies.

### 3.1. Traditional Network Technology

Traditional network technologies include wireless networks and campus networks, which not only provide the basic environment for platform operation, but also provide the connection hub for the IoT layer and the resource and data center layers.

### 3.2. IoT Technology

The main function of the Internet of Things technology is to support the dynamic collection of platform data and use terminal sensors to collect various data of physical education activities in colleges and universities, for example, the position, posture, and movement trajectory of each student in football teaching; the starting position and posture of each student in track and field teaching; each student's shooting posture, movement trajectory, basketball speed, and movement trajectory in basketball teaching. The platform is equipped with sensors to collect teaching process data and transmit the data to the resources and data center layers through the wireless network layer and the campus network layer.

### 3.3. Mobile Terminal Technology

The commonly used mobile terminal technology is Android. This technology provides support for the real-time interaction of the physical education platform in colleges and universities and is conducive to the dynamic real-time interaction between teachers and students. In addition, the teacher can also send the students' classroom learning situation to the students through the mobile terminal, for example, the time, speed, ranking, blood pressure, heart rate, and other indicators of each student in the 1000-meter long-distance running.

### 3.4. Data Mining Technology

With the advancement of teaching activities, the data collected by the platform for physical education activities in colleges and universities continues to increase. Through data mining technology, a large amount of data can be mined and analyzed, so as to obtain an objective and detailed understanding of the specific conditions of physical education activities in colleges and universities. For example, through comparative analysis of the physical fitness test data of college students every semester, the existing deficiencies are found, and then a reasonable exercise plan is formulated for the students. This plays an important guiding role in grasping the laws of physical education teaching in colleges and universities, grasping the learning situation of students, carrying out physical education activities in colleges and universities in a targeted manner, and guiding college students to exercise independently.

### 3.5. Modeling and Simulation Technology

Modeling and simulation technology is to use the collected data and theoretical data to establish a computer-implemented simulation model for physical education activities through modeling and simulation software. Models that can be driven by actual data are designed to reflect the real situation of actual physical education activities and to be intuitively recognized and understood by the participants of the teaching activities. In this way, as the main body of physical education activities, participants can summarize and ask questions from the perspective of the main body and formulate correction methods. It can be said that the simulation model is a computer restoration of data-driven real physical education activities, and it is a link platform and participants bridge.

### 3.6. Database Technology

Database technology is a relatively mature technology, which realizes the data storage task of the entire platform and is also the bearer of the data warehouse of data mining.

### 3.7. Application Development in Combination with Physical Education Needs

**Figure 3:**
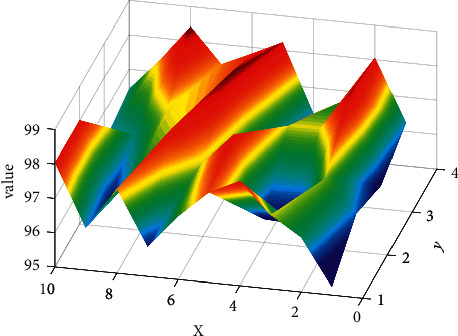
Predicted value.

If the above platform-related technologies mostly rely on existing IT technologies and products, this part of the application development technology is biased towards research and development. The requirements are to design the technical realization ideas of each link and finally use the existing IT technology, such as JAVA.net and other development technologies, to develop the entire platform, which is equivalent to the specific implementation of the entire platform, connecting the above technologies. It is also the specific implementation of the services provided by the platform and is the core of the entire platform. The predicted data is shown in Figure 3.

The Internet of Things technology and mobile terminal technology correspond to the “movement points” in physical education activities and are the original collection layer of sports. The network and database are the storage carriers of the “movement points” data. The modeling and simulation technology is the reproduction of the laws of physical education activities. Data mining technology is the display of macroscopic characteristics of a large amount of data. In short, it is necessary to closely combine IT technology and physical education needs to design an advanced teaching platform.

## 4. Simulation Test

**Figure 4:**
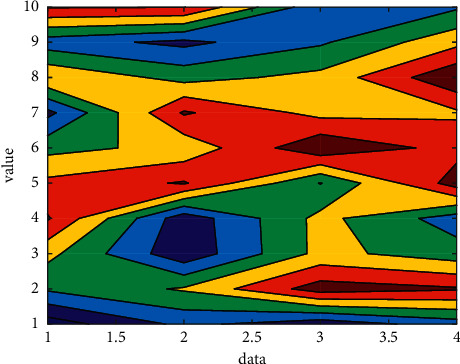
Prediction.

The teacher's course scheduling module is used to realize the teacher's course scheduling, and its main functions include adding, deleting, and modifying. When the teacher completes the entry of one physical education course information, he can complete the entry of the next physical education course information through “Add”; when the entry is wrong, he can operate the wrong information through “Delete” or “Modify.” Generally speaking, the arrangement of physical education courses is a combination planning problem of time, teachers, and students. In the Internet of Things environment, the arrangement of physical education courses cannot conflict in terms of time, teachers, and students. For example, a student cannot take two classes in the same time period; the same teacher cannot take two classes at the same time; each student is not allowed to attend classes for more than 2 hours per week. The prediction is shown in Figure 4.

The student course selection module manages the students' selection of physical education courses, and its main functions include deleting and statistics of students' course selection information. When the student does not want to take the selected sports course, delete the selected course through the “Delete” function. Since some colleges and universities require each student to have 6 credits of physical education courses, students need to choose multiple physical education courses that do not conflict with each other in time and view the selected course information through the “Statistical Student Course Selection Information” function. This module completes the management of elective courses and can also count the number of applicants for elective courses. When the number of physical education courses is full, it is necessary to determine the number of students who choose this physical education course according to the time of students' course selection.

The student basic information setting module is mainly responsible for managing the personal information of all registered students in the school. The main functions include adding, deleting, modifying, and searching for student information. When a student completes a piece of information, they can enter the next piece of information through “Add”; when a student enters wrong information, they can use “Delete” or “Modify” to carry out the next step for the wrong information. After completing the entry of all the information, it is necessary to further check the previously entered information through “Find Student Information.” Each student has his own student number. After the administrator adds a new student, the new student can directly log in to the sports course management system to browse personal information.

The interactive communication module realizes the information interaction among teachers, students, and administrators, and the communication is mainly realized in the form of online forum posts. Students and teachers can post or view posts, and administrators can manage posts posted by teachers or students in addition to managing student and teacher users. Therefore, the interactive communication module contains not only the user management data table, but also the post management data table. The user management data table mainly manages the user's name, gender, grade, student ID, and other information to obtain data; the post management data table is mainly for the title, content, publication date, publisher's name, gender, grade, student ID of the post published by the user, and other information to implement management to obtain data.

The experiment was arranged in April 2018, testing the third-year computer majoring physical education teachers and 300 students in a university in Beijing, and using the system of this paper to complete the scheduling and selection of physical education courses. The experimental test was completed in the computer room of the university. The operating system of the computer is Fedora 13 Linux. The program debugging tool GDB 7.0.1 was used to test the results of the system's course scheduling and course selection monitoring for teachers and students.

### 4.1. Analysis of Physical Education Course Arrangement

**Figure 5:**
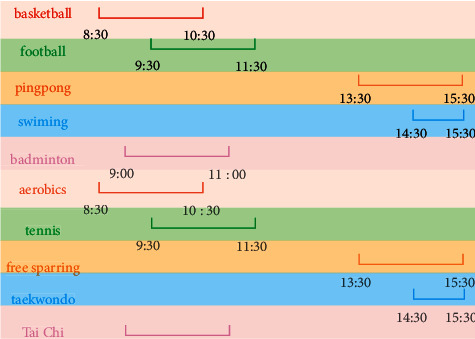
Course arrangement.

The system in this paper mainly provides the course items, time, credits, and other contents of the school's public physical education courses. Students choose physical education courses according to their personal circumstances, and the total number of applicants for each physical education course is 200. The course arrangement of physical education teachers is shown in Figure 5.

It can be seen from Figure 3 that the physical education teachers have set up 10 physical education courses such as basketball, table tennis, football, synchronized swimming, badminton, and free fighting using the system in this paper. 09:00–11:00, 09:30–11:30, 13:30–15:30, and 14:30–16:30 are five time periods, 2 hours per week; each physical education course's credits are 2 credits, the total number of applicants for each sports course is 200, and the remaining number of basketball courses is at most 142, indicating that the number of students who choose basketball courses is the least, followed by the number of students who choose football courses, table tennis courses, synchronized swimming courses, badminton courses classes, aerobics classes, tennis classes, and taekwondo which classes have the highest number of students.

### 4.2. Monitoring and Analysis of Students' Course Selection

**Figure 6:**
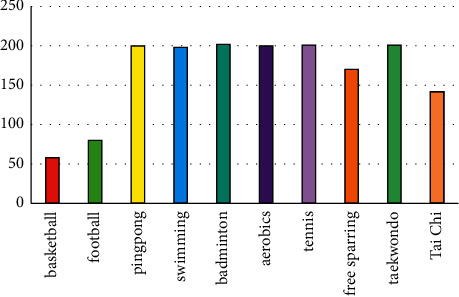
The number of students enrolled in each physical education course.

Using the system in this paper to monitor the number of students enrolled in each physical education course at the same time, the results are shown in Figure 6.

Figure 6 shows the students' selection of courses monitored by the system: badminton, tennis, taekwondo, aerobics, table tennis, and synchronized swimming are the most popular among students, and all 200 course selection places are full. Popular with students, 170 students and 142 students have chosen kickboxing courses and tai chi courses, respectively; basketball and football have the least number of courses, 58 and 80, respectively. It can be seen that college students' preference for sports has changed from the traditional single basketball and football to the trendy sports such as taekwondo, aerobics, and synchronized swimming.

### 4.3. Course Information Analysis

**Figure 7:**
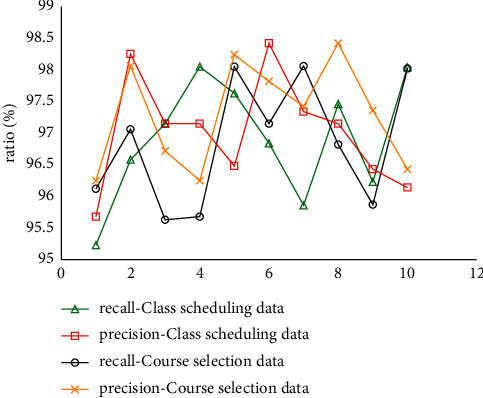
Statistics.

The statistical results of the analysis performance of the system in this paper on the scheduling and selection data of different physical education courses are shown in Figure 7.

**Figure 8:**
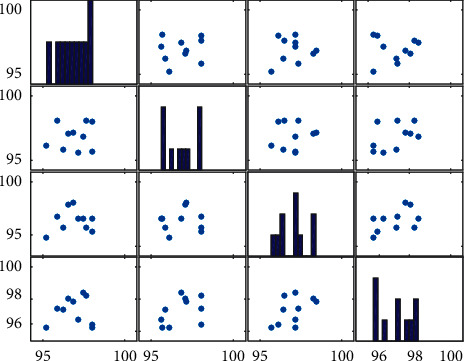
Evaluated data.

From the statistics in Figure 7, it can be concluded that the recall rate and precision rate of the 10 kinds of physical education course scheduling data and course selection data systematically analyzed in this paper are higher than 95%, and the analysis performance is excellent. The evaluated data are shown in Figure 8.

## 5. Conclusion

This paper designs a sports course management system based on the Internet of Things to realize the intelligent management of sports courses, which is mainly reflected in the following aspects:

1. On the application side, the system realizes teachers' course scheduling, students' course selection, and teacher-student communication through modules such as teacher course scheduling, student course selection, and interactive communication, and realizes intelligent management of physical education courses.
2. The system realizes the management and information maintenance of college physical education course information through the management functions and maintenance functions in the functional structure and can accurately analyze the performance of physical education courses, reasonably arrange the physical education curriculum according to the results, and enhance the students' participation in physical education course activities—positivity.
3. The system builds a physical education course information management model based on the Internet of Things in the infrastructure and server to realize the perception, transmission, sharing, and query of physical education course information.
4. Through the online forum in the interactive communication module, teachers can communicate with students about course feedback and improve the interactivity of physical education course information management.

## Data Availability

The data used to support the findings of this study are available from the author upon request.
